# Distinct Characteristics of Patients With Fragility Hip Fracture in Greece: Evidence From the Greek National Fragility Hip Fracture Registry Utilizing the Minimum Common Data Set

**DOI:** 10.7759/cureus.103990

**Published:** 2026-02-20

**Authors:** Efthymios Iliopoulos, Theo Tosounidis, Reichan Molla Mustafa, Athanasia Charmpi, Fotios Tilkidis, Alkison Kotsis, Dimitra Melissaridou, Maria Sentona, Georgios Graikos, Androniki Kyprianou, Irini Tatani, Ioannis Gkiatas, Byron Chalidis, Ioannis V Papachristos, Christiana Zidrou, Olga Savvidou, Georgios Drosos

**Affiliations:** 1 Orthopaedics, University General Hospital of Alexandroupolis, Democritus University of Thrace Medical School, Alexandroupolis, GRC; 2 Trauma and Orthopaedics, University Hospital of Heraklion, University of Crete Medical School, Heraklion, GRC; 3 Orthopaedics and Traumatology, General Hospital of Alexandroupolis, Democritus University of Thrace Medical School, Alexandroupolis, GRC; 4 Orthopaedics, Patras General University Hospital, University of Patras School of Medicine, Patras, GRC; 5 Orthopaedics, General University Hospital of Ioannina, University of Ioannina Medical School, Ioannina, GRC; 6 Orthopaedic Surgery, University Hospital of Heraklion, University of Crete Medical School, Heraklion, GRC; 7 Orthopaedics, ‘Attikon’ General University Hospital, National and Kapodistrian University of Athens, Athens, GRC; 8 Orthopaedics, Papageorgiou General Hospital, Thessaloniki, GRC; 9 Orthopaedics and Traumatology, General Hospital of Thessaloniki Georgios Papanikolaou, Thessaloniki, GRC; 10 Orthopaedics, Tzaneio General Hospital of Piraeus, Piraeus, GRC; 11 Stavros Niarchos Foundation Complex Joint Reconstruction Center, Hospital for Special Surgery, New York, USA; 12 Orthopaedics, Aristotle University of Thessaloniki School of Medicine, Thessaloniki, GRC; 13 Orthopaedics, KAT Hospital, Athens, GRC; 14 Orthopaedics, General Hospital of Thessaloniki “Papageorgiou”, Thessaloniki, GRC

**Keywords:** elderly trauma, fragility fractures, fragility hip fractures, geriatric hip fracture, hip fracture, length of stay, mortality, registry

## Abstract

Background

Over the past two decades, fragility hip fracture registries have been implemented in various countries worldwide. These registries have played a key role in documenting the state of individual health care systems, highlighting the specific challenges they face, and showcasing the unique characteristics of each population. The aim of the present study is to highlight the distinct characteristics of the Greek fragility hip fracture patients, utilizing the data from the first two years of the first national fragility hip fracture registry in Greece.

Materials and methods

Nine orthopaedic departments from eight hospitals across the country participated in this prospective multicenter study. All cases of hip fragility fractures between September 2022 and December 2024 were prospectively recorded in a centralized database. Data collection was based on the 21-point Minimum Common Data Set recommended by the Global Fragility Fracture Network, with the addition of 30-day mortality data. A complete case analysis was performed, excluding records with missing data. Multivariable regression and neural network models were developed to assess predictors of 30-day mortality and length of stay. Model discrimination was assessed using the area under the receiver operating characteristic curve (AUC).

Results

A total of 2139 patients (aged 82.4±8.4 years) with a fragility hip fracture admitted in the involved orthopaedic departments were included in the study. The majority of the patients were female (70,6%) and had sustained a peri-trochanteric fracture (58.2%). A significant proportion of the patients were staying at their own home before the injury, and 65% of the patients were fully independent, mobilising freely or with one stick outdoors. Most of the patients were treated surgically (94.4%), but the surgical intervention was performed within the first 48 hours in only 31% of the cases. The mean in-hospital length of stay was 10.68 ±8.1 days, with 60% of the patients returning to their own home after discharge. The in-hospital mortality was 4%, while the 30-day mortality reached 10.9%.

Multivariate and neural network analysis revealed that increased age and male gender negatively affect the 30-day mortality of these patients. Their pre-injury residence as well as the exit destination were also significant predicting factors for the 30-day mortality. The prompt surgical intervention (within 48 hours of admission) and the pre-injury mobility level influence the length of in-hospital stay of these patients (p<0.001).

Conclusions

Greece’s first national hip fracture registry highlights key healthcare system gaps, particularly the low rate of timely surgeries for fragility hip fractures, which is linked with prolonged hospital stay. Despite this, the 30-day mortality rate aligns with other European countries. High pre-injury independence and the number of patients discharged home positively influence their outcomes.

## Introduction

Over the past two decades, fragility hip fracture registries have been established in numerous countries globally, serving as essential tools for evaluating healthcare system performance and establishing a unified benchmarking system. These registries have contributed significantly to identifying system-specific challenges and delineating the distinct epidemiological and clinical characteristics of national populations [1-3]. Throughout the years, such registries have contributed to improvements in local healthcare systems, including reductions in both the time to surgery and patient mortality rates [4,5].

Pioneering registries, such as the National Hip Fracture Registry in the United Kingdom, along with those in the United States, Australia, and New Zealand, and the Scandinavian countries, have now reached a mature stage. These registries are actively identifying key issues and providing data-driven recommendations [1,2,4-7]. While their findings can inform national policies and support country-specific conclusions, direct comparisons between registries have proven to be unreliable and, in some cases, not feasible [8]. This issue has become increasingly evident in recent years, as new registries are being established in new countries, each with distinct healthcare systems and population characteristics, which face different challenges [3,9,10]. The Global Fragility Fracture Network (FFN) has introduced the Minimum Common Data Set, which is a standardized set of 21 variables aimed at harmonizing data collection across different registries and promoting international collaboration in healthcare audits [11].

This study presents the results from the first two years of Greece’s national fragility hip fracture registry, established by the FFN Greek Chapter (FFN-Gr) [12]. It describes key features of the Greek healthcare system and elderly population, identifies local challenges in hip fracture care, and compares the findings with data from other countries.

Preliminary results of this article were previously presented as a wall poster at the 13^th^ Fragility Fracture Network Global Congress, October 2-4, 2025, Porto, Portugal.

## Materials and methods

Following an initiative of the FFN Gr [12], a nationwide, multicentre collaborative group was established with the aim of developing the first national registry for fragility hip fractures in Greece. Within this framework, nine orthopaedic departments from eight hospitals distributed across different geographic regions of the country participated in the present study, ensuring broad national representation of clinical practice and patient demographics.

Study population

Eligibility for inclusion in the registry and the present analysis was based on admission to one of the participating orthopaedic departments due to a low-energy fragility fracture of the proximal femur. Fragility fractures were defined as fractures resulting from a fall from standing height or less, or occurring in the absence of significant trauma. Patients were excluded if the fracture resulted from high-energy mechanisms, if they sustained polytrauma, if they were younger than 60 years of age, or if the fracture was considered pathological in nature (e.g., secondary to malignancy).

All consecutive patients presenting with fragility fractures of the hip and treated at the participating centres between September 2022 and December 2024 were prospectively identified and enrolled. Data collection was conducted in a prospective manner at each site by designated members of the orthopaedic or multidisciplinary care teams. To ensure data protection and patient confidentiality, all collected data were anonymised prior to entry and stored in a centralized, secure electronic database accessible only to authorized study personnel.

Data collection

Data collection was standardized across all centres using the 21-point Minimum Common Data Set (MCDS) recommended by the FNN [11]. This dataset includes key variables related to patient demographics, patients’ pre-injury status, fracture characteristics, perioperative management, and hospitalization data, allowing for consistency and comparability both nationally and internationally. More specifically, surgery within 48 hours was calculated from the admission of the patient to the hospital. Mobilisation within 24 hours was calculated from the time the operation ended, was assessed by the physiotherapists in the orthopaedic wards, and was defined as positive when the patient managed to sit out of bed. Pressure ulcers were diagnosed and classified using the European Pressure Ulcer Advisory Panel (EPUAP)/National Pressure Ulcer Advisory Panel (NPUAP) classification system.

Only new ulcers developed during hospitalisation were reported, while pre-existing ulcers were documented but excluded from the analysis. Discharge destination is categorized into mutually exclusive groups: own home, nursing home, inpatient rehabilitation, transfer to another acute hospital, or in-hospital death. Each patient was assigned to a single discharge category. In addition to the standard MCDS variables, the 30-day mortality was recorded as an outcome measure, given its recognized importance as a quality indicator in hip fracture care, via the national civil registry linkage and hospital records, with the preferable end point at 30 days post surgery. In-hospital deaths were included in the 30-day mortality models to avoid immortal time bias.

Ethics

Ethical approval was obtained from the institutional review board of each participating center. The coordinating center approval was granted by the University General Hospital of Alexandrouplis Scientific Ethical Board (approval number ES19/Th21/6-10-2022). Informed consent was waived due to the observational registry design. The study was performed in accordance with the ethical standards as laid down in the 1964 Declaration of Helsinki.

Statistical analysis

Data from the central database were analysed using the IBM SPSS Statistics for Windows, version 29.0 (IBM Corp., Armonk, New York, United States). A significance level of 0.05 was applied for all statistical tests. The same program was used to perform univariate analyses (chi-square test, independent samples t-test, and one-way ANOVA, Mann-Whitney U test) and multivariate models (logistic and multiple regression), as well as neural network-based machine learning factor analysis to evaluate 30-day mortality and in-hospital length of stay. Continuous variables were assessed for normality using the Shapiro-Wilk test and visual inspection of histograms. Normally distributed variables were analyzed using parametric tests, while non-normally distributed variables were analyzed using non-parametric alternatives. Assumptions of linear regression, including normality of residuals and homoscedasticity, were evaluated.

## Results

A total of 2139 patients with a fragility hip fracture admitted to the involved orthopaedic departments were included in the study. The majority of the patients were female (70.6%, n=1510) at a mean age of 82.4 ±8.4 years. Almost one quarter of the patients (22.3%, n=478) had known dementia at the time of admission. A significant proportion of the patients (95%, n=2006) were staying at their own home before the injury, and 65% (n=1349) of the patients were fully independent, mobilising freely or with one stick outdoors. Most of the patients had peri-trochanteric hip fractures (58,2%, n=1245), while less frequent were the intracapsular hip fractures. (Table 1).

**Table 1:** Descriptive data of the cohort and respective monovariant analysis for 30-day mortality and length of stay. * indicates statistical significance; Statistical tests used: x^2^ & Mann-Whitney U Test. Percentage values were calculated after excluding missing values from the total number of enrolled patients (N=2139). Percentage values of the missing values were calculated from the total number of enrolled patients (N=2139) and are indicated in brackets. ASA: American Society of Anesthesiologists; LOS: length of hospital stay

Variables			Frequency	Percentage	p-value for 30-day mortality	p-value for LOS
Sex		Female	1510	70.6	<0.001*	0.73
		Male	629	29.4		
Age (Years)			82.38	±8.43	<0.001*	0.048*
Pre-Injury Residence	Own Home		2006	94.5	<0.001*	0.7
	Nursing Home		93	4.4		
	Hospital		12	0.6		
	Rehabilitation Centre		4	0.2		
	Unknown		7	0.3		
	Missing Values		17	(0.8)		
Pre-Injury Mobility	Free		838	39.8	<0.001*	0.02*
	Outside with 1 stick		511	24.3		
	Outside with 2 sticks		301	14.3		
	Inside only – Never Outside		392	18.6		
	No Mobility		32	1.5		
	Unknown		30	1.4		
	Missing Values		35	(1.6)		
Mental State	Normal		1368	64.0	<0.001*	0.007*
	Known Dementia		478	22.3		
	Not-Known Dementia but Positive Tests		249	11.6		
	Missing Values		44	(2.1)		
ASA Grade (2.72±0.87)	I		102	4.8	<0.001*	<0.001*
	II		832	39.4		
	III		784	37.1		
	IV		345	16.3		
	V		48	2.3		
	Missing Values		28	(1.3)		
Fracture Type	Intracapsular		861	40.25	-	-
	Peri-trochanteric		1245	58.20		
	Other & Missing Values		33	1.5		

Most of the patients were treated surgically (94,4%, n=1940), with a mean ASA (American Society of Anesthesiologists) grade of 2.72 ±0.87. The surgical intervention was performed within 48 hours of their admission to the hospital in only 31% (n=585) of the cases, mostly under spinal anaesthesia (75.5%, n=1461), while the rest were under general anaesthesia. Most of the extracapsular fractures were treated by using an intramedullary nail (91.5%, n=1116), and most of the patients with intracapsular fractures were treated with hip hemiarthroplasty (80.6%, n=675) (Table 2).

**Table 2:** Injury and operative data of the cohort and respective monovariant analysis for 30-day mortality and length of stay. * indicates statistical significance; statistical tests used: x2 & Mann-Whitney U Test. Percentage values were calculated after excluding missing values from the total number of enrolled patients (N=2139). Percentage values of the missing values were calculated from the total number of enrolled patients (N=2139) and are indicated in brackets. LOS: length of hospital stay

Variables		Frequency	Percentage	p-value for 30-day mortality	p-value for LOS
Fracture Type	Undisplaced Intracapsular	80	3.7	0.71	0.17
	Displaced Intracapsular	781	36.6		
	Intertrochanteric	1121	52.5		
	Subtrochanteric	124	5.8		
	Other	30	1.4		
	Missing Values	3	(0.1)		
Type of Surgery	Non-Operative Management	114	5.6	0.03*	<0.001*
	Cannulated Screws	10	0.5		
	Dynamic Hip Screw (DHS)	11	0.5		
	Intramedullary Nailing	1124	54.7		
	Hip Hemiarthroplasty	688	33.5		
	Total Hip Arthroplasty	71	3.5		
	Other	36	1.8		
	Missing Values	85	(4)		
Type of Surgery for Intracapsular Fractures	Hip Hemiarthroplasty	675	80.6	-	-
	Total Hip Arthroplasty	70	8.4		
	Other	93	11		
	Missing Values	23	(2.7)		
Type of Surgery for Peri-Trichanteric (Inter- + Sub- Trochanteric) Fractures	Dynamic Hip Screw (DHS)	8	0.7	-	-
	Intramedullary Nailing	1116	91.5		
	Other	97	7.8		
	Missing Values	24	(1.9)		
Surgery within 48 hours from admission	Yes	585	30.9	0.07	<0.001*
	No	1308	69.1		
	Missing Values	246	(11.5)		
Type of Anaesthesia	General Anaesthesia	464	24.0	0.09	0.2
	Spinal Anaesthesia	1461	75.5		
	Other	8	0.4		
	Missing Values	206	(9.6)		

The mean in-hospital length of stay (LOS) was 10.68 ±8.1 days, with 13.2% (n=283) developing a new pressure ulcer during hospitalisation. LOS was non-normally distributed and is reported as median with interquartile range (IQR) in Table 3; median LOS was nine days (IQR 0-132). Group comparisons were performed using the Mann-Whitney U test. Internal medicine doctors supported the Orthopaedic patients during the more acute hospitalisations in 59% (N= 1233) of the cases. Unfortunately, the number of Geriatric patients was extremely low (only 1.7%), as in Greece, the Geriatric and Orthogeriatric subspecialties are yet to be officially recognised. Only 54.2% (n=1096) of the patients managed to mobilise outside of the bed on the first postoperative day. The most frequent discharge destination was the patients’ home (59%, n=1233), followed by a rehabilitation centre (30.3%, n=635). Unfortunately, only about one fourth of the patients receive anti-osteoporotic medication at discharge (26.6%, n=545). The in-hospital mortality was 4% (n=84); the mortality rate was significantly higher for the patients discharged to rehabilitation clinics compared to those discharged to their own home (p<0.001). Of 2139 patients in the registry, 30-day mortality status was available for 1612 (75.4%). Among patients with complete follow-up, 175 deaths occurred, corresponding to a 30-day mortality of 10.9% (n=175) (Table 3).

**Table 3:** Hospitalisation and follow-Up Data of the cohort and respective monovariant analysis for 30-day mortality and length of stay. * indicates statistical significance; statistical tests used: x2 & Mann-Whitney U Test. Percentage values were calculated after excluding missing values from the total number of enrolled patients (N=2139). Percentage values of the missing values were calculated from the total number of enrolled patients (N=2139) and are indicated in brackets. Length of Stay (LOS) variable was not normally distributed, so it is presented as mean±SD, median (IQR)

Variables	Frequency	Percentage	p-value for 30-day mortality	p-value for LOS
New Pressure Ulcer			-	-
Yes	283	13.2		
No	1741	81.4		
Missing Values	115	(5.4)		
LOS (Days), mean±SD, median (IQR)	10.68±8.11, 9 (0-132)		0.27	-
Medical Support			-	-
Internal Medicine	1233	59.61		
Geriatrics	35	1.7		
None	764	36.6		
Other	54	2.6		
Missing Values	53	(2.5)		
Mobilisation within 24 hours of Surgery			<0.001*	0.5
Yes	1096	54.2		
No	824	40.7		
Missing Values	219	(10.2)		
In-Hospital Mortality			-	-
Yes	84	4.0		
No	2020	96.0		
Missing Values	35	(1.6)		
Exit Destination			<0.001*	0.08
Own Home	1233	58.9		
Nursing Home	57	2.7		
Rehabilitation Centre	635	30.3		
Unknown	46	2.2		
Death	84	4.0		
Missing Values	84	(3.9)		
Discharge Osteoporosis Medication			-	-
Start	343	16.8		
Continued Same	155	7.6		
Changed	47	2.3		
(Subtotal – Patients with anti-osteoporotic medication at discharge)	545	26.6		
Stopped	3	0.1		
Unknown	1499	73.2		
Missing Values	92	(4.3)		
30-Day Mortality			-	0.27
Yes	175	10.9		
No	1437	89.1		
Missing Values	527	(24.6)		

Univariate analysis revealed that the age, ASA grade, gender, mental state, the type of residence pre-injury, the level of independence, the conservative management, the mobilisation on the first postoperative day, and the exit destination significantly affected the 30-day mortality of the patients (p<0.001). The length of stay had a positive correlation with increased age and ASA grade (p<0.05). It was also significantly affected by the pre-injury mobility levels, known dementia, type of surgery (with conservative management and cannulated hip screws having significantly less LOS), and the timing of surgery (p=0.02 - <0.001).

**Figure 1:**
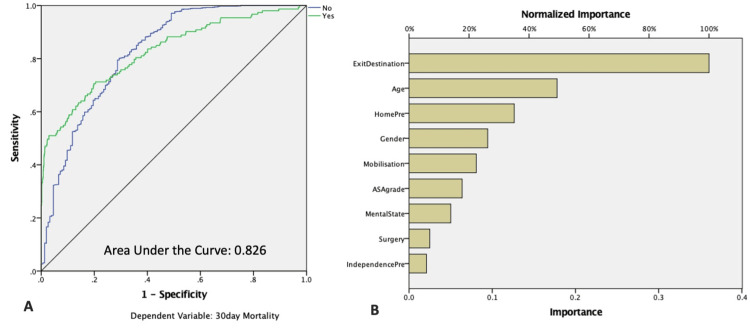
Neural Network analysis for the 30-day mortality. A: ROC curve analysis; B: Bar chart for neural network analysis

Multivariate analysis model with the 30-day mortality as dependent variable revealed that only age, male sex, pre-injury residence, and discharge destination were the only variables that maintained their significant influence on the 30-day mortality (Table 4). Neural network analysis for the 30-day mortality confirmed these results by reaching a good predictive value with an AUC of 0.826. The discharge destination and the age were the most important predictive variables (Table 4 and Figure 1).

**Table 4:** Multivariate and neural network analysis with the 30-day mortality as independent variable. * indicates statistical significance; Statistical Analysis: Binary Logistic Regression

Variables	Sig. (p)	Beta	OR (95% C.I.)	Independent Variable Importance	Normalised Importance (%)
Age (Years)	<0.001*	1.080	1.045 – 1.118	0.178	49.3
ASA grade	0.115	1.273	0.943 – 1.719	0.064	17.7
Gender	<0.001*	0.406	0.261 – 0.632	0.095	26.2
Mental State	0.979	0.94	0.50 – 1.76	0.050	13.9
Pre-Injury Residence	<0.001*	0.287	0.156 – 0.531	0.126	35.1
Pre-Injury Mobility	0.599	1.150	0.683 – 1.937	0.021	5.8
Surgery	0.521	1.963	0.251 – 15.369	0.025	6.9
Mobilisation within 24 hours of Surgery	0.385	5.901	0.46 – 73.44	0.081	22.4
Exit Destination	<0.001*	0.367	0.227 – 0.594	0.360	100

Multiple imputation using fully conditional specification was performed to address 24.6% missing mortality data (30 imputations). In pooled logistic regression analysis, age (OR 1.06 per year, p<0.001), gender (OR 2.17, p<0.001), pre-admission residence (OR 2.20, p=0.006), and discharge destination (OR 2.12, p<0.001) were independently associated with the 30-day mortality. Other variables were not statistically significant after adjustment (Table 5).

**Table 5:** Multivariable logistic regression analysis of factors associated with 30-day mortality after multiple imputation of missing outcome data. 30-day mortality data were missing in 24.6% of cases and were addressed using multiple imputation by fully conditional specification (30 imputations). Estimates represent pooled adjusted odds ratios (OR) with 95% confidence intervals (CI) calculated using Rubin’s rules. All variables listed were included simultaneously in the model. ASA: American Society of Anesthesiologists

Variable	Adjusted OR	OR (95% CI)	p-value
Age (per year)	1.06	1.029 – 1.085	<0.001*
ASA Grade	1.31	0.983 – 1.740	0.065
Gender	2.17	1.486 – 3.178	<0.001*
Metal State	0.94	0.726 – 1.215	0.634
Pre-Injury Residence	2.20	1.259 – 3.834	0.006*
Pre-Injury Mobility	1.024	0.686 – 1.528	0.909
Surgery	0.57	0.240 – 1.362	0.206
Mobilisation within 24 hours of Surgery	0.88	0.619 – 1.244	0.462
Exit Destination	2.12	1.841 – 2.444	<0.001*

**Figure 2:**
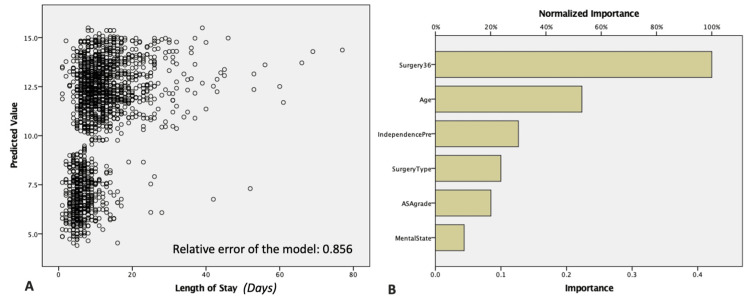
Neural Network analysis for the length of stay. A: Scatter plot for neural network analysis; B: Bar chart for neural network analysis

The multivariate model for the length of stay as the dependent variable revealed only the prompt surgical intervention and the pre-injury mobility status as the only significant factors (Table 6). Neural network analysis confirmed that surgery within 48 hours of admission was the most significant factor affecting the prolonged hospital stay (Table 6 and Figure 2).

**Table 6:** Multivariate and neural network analysis with the length of stay as independent variable. * indicates statistical significance; Statistical Analysis: Linear Multiple Regression ASA: American Society of Anesthesiologists

Variables	Sig. (p)	Beta	OR (95% C.I.)	Independent Variable Importance	Normalised Importance (%)
Age (Years)	0.098	-0.042	-0.080 – 0.007	0.223	53
ASA grade	0.661	-0.010	-0.495 – 0.314	0.085	20.1
Pre-Injury Mobility	0.001*	0.084	0.544 – 2.133	0.127	30
Mental State	0.937	0.002	-0.496 – 0.538	0.044	10.4
Type of Surgery	0.823	-0.005	-0.506 – 0.402	0.100	23.7
Timing of Surgery	<0.001*	-0.393	-7.095 – -5.704	0.421	100

## Discussion

The establishment of the Fragility Hip Fracture Registry in Greece, led by the FNN-Gr in recent years, has shed light on the challenges facing the Greek healthcare system and offered valuable insights into the conditions within public hospitals [9]. This study highlights several key and distinct characteristics of elderly patients suffering from fragility hip fractures in Greece. Although the majority of these patients do not undergo surgery promptly, the 30-day mortality rate remains relatively moderate at approximately 11%. Factors such as age, gender, and place of residence appear to influence mortality outcomes. Notably, delay in surgery emerged as the primary contributor to prolonged hospital stay, pointing to a clear area for targeted improvement.

Patients from different countries vary in demographics, comorbidities, and fracture types; consequently, healthcare professionals in each system face different challenges, accounting for the local cultural factors as well. In the Greek cohort, the mean age was approximately 82.5 years, comparable to that in several Scandinavian and Central European countries such as Sweden, Denmark, Norway, the United Kingdom, Germany, France, and the Netherlands (82-85 years). However, it was lower than in some Mediterranean countries like Italy and Spain, where the mean age ranges from 86 to 87 years [8,13-15]. In Greece, over 70% of patients were female, a proportion similar to those observed in the United Kingdom, Ireland, and Germany (69-72%). Sweden reported slightly lower rates (66%), while Italy and Spain had higher rates (76-77%). A significant majority (95%) of Greek patients were living independently at home prior to their injury, markedly higher than the European average of 71-75%, and even higher than the higher reported percentages of the United Kingdom, Ireland, and Italy (81-91%) [8,15]. Pre-injury mobility levels in Greece were also comparable to those in other European countries, with about 65% of patients able to walk unaided or with a single walking stick outdoors. The mean ASA score in the Greek cohort was 2.7, with only 55.7% of patients classified as ASA grade III or above. This is noticeably lower than reported figures across Europe, which range from 61% to 74.5% [8,15,16]. Cognitive impairment (either diagnosed dementia or positive cognitive screening) was present in 34% of Greek patients, aligning with the European range of 17% to 44% [8]. Usually, intracapsular hip fractures are more frequent in this type of patient cohort compared to peritrochanteric ones [8]. This proportion is opposite in the Greek and Spanish cohort, with peri-trochanteric fractures reaching almost 60% of hip fragility fractures [8,10,16].

According to a review of European annual reports of hip fracture registries by Werner et al., healthcare systems manage the burden of fragility hip fractures in diverse ways, influenced by local policies, available funding, human resources, and clinical expertise [8]. In Greece, conservative treatment for fragility hip fractures was applied in 5.6% of cases. This rate sits at the higher end of the European spectrum (1-5%), with Ireland reporting a comparable rate of 5%. For intracapsular femoral neck fractures, the most commonly performed procedure in Greece was hip hemiarthroplasty, accounting for 33.5% of cases. In contrast, total hip arthroplasty and cannulated screw fixation were rarely used, at 3.5% and 0.5%, respectively. This treatment pattern is also observed in Spain and the Netherlands. However, other European countries, including Italy, Sweden, and Denmark, show a greater preference for total hip arthroplasty, with rates exceeding 10%. Cannulated screw fixation is more commonly used in Scandinavian countries, where it accounts for 10-15% of treatments. For peritrochanteric fractures, the intramedullary proximal femoral nailing is the preferred method in Greece, used in nearly 55% of cases. The dynamic hip screw (DHS) is rarely chosen, appearing in only 0.5% of cases. A similar preference for intramedullary nails is observed in Germany, Italy, and Spain. Conversely, Ireland and Scandinavian countries report higher DHS usage (15-20%), although IM nails remain the dominant choice. In contrast, DHS is more frequently used in Scotland and England. In terms of anaesthesia, approximately 75% of surgeries in Greece are performed under spinal, a pattern similar to that of Ireland, Italy, and Norway. Sweden and Spain favour spinal anaesthesia even more, with usage rates above 90%, while Germany predominantly uses general anaesthesia (94%). Countries like England, Scotland, and the Netherlands show a more balanced use of both techniques [8].

One significant weakness of the Greek healthcare system is the delayed surgical intervention, with only 30% of patients undergoing surgery within 48 hours of admission, substantially lower than the European average, which ranges from 48% to 95% [8,16]. Additionally, only 55% of patients in Greece were mobilised out of bed on the first postoperative day, again falling short of the European range of 67-82%. This delay in mobilisation contributed to a higher incidence of pressure sores, present in 13% of Greek patients, compared to 3-4.8% in countries such as England, Germany, Italy, and Spain [8]. Approximately 25% of Greek patients were discharged with anti-osteoporotic medications, a rate comparable to Italy and the Netherlands but significantly lower than in countries like England, Ireland, and Denmark. Notably, around 60% of patients in Greece were discharged directly to their homes, the highest percentage reported in Europe, where rates range between 12% and 52% [8,16].

In the Greek cohort, in-hospital mortality was recorded at 4%, while 30-day mortality reached 10.9%. The in-hospital mortality rate aligns with those reported across other European countries, which range from 1.5% to 6%. However, the 30-day mortality rate in Greece was slightly higher than the European average, which falls between 5.5% and 9.5% [8]. Among the variables examined, only increased age, male gender, pre-injury living situation, and discharge destination were found to have a significant impact on mortality. These findings are in line with other registries, such as the Spanish, in which the same factors affect the mortality rates significantly. The Spanish registry reports significant influence by the higher ASA grade and time to surgery more than 48 hours, as well as variables that are not significant in the Greek cohort [16]. Age, gender, ASA grade, cognitive status, and residential status are significant predictor factors for mortality in the Dutch registry, also [17,18]. Although delayed surgery has been shown in cohorts from other countries to be a major contributing factor to higher mortality rates, this association was not observed in the Greek population [19]. The low rates of seeker patients (ASA grade III and above) may have positively affected the mortality rate, counteracting the delays in surgery. Interestingly, only a weak correlation between 30-day mortality and delay in surgery was reported recently from the Spanish registry [16].

The average hospital stay for patients in the Greek cohort was just over 10 days, comparable to durations reported in Scotland, Denmark, Italy, and Spain. In contrast, significantly shorter hospital stays are reported in Sweden, Finland, and the Netherlands, where the average ranges from 4.2 to seven days. On the other end, England, Germany, and Ireland report notably longer hospitalisations, ranging from 15 to 19.5 days [8,16]. Interestingly, the early mobilisation of the patients after surgery had no significant influence on the LOS in the present study, which contradicts the findings of other studies [20]. In Greece, the timing of surgery and the patient’s pre-injury mobility appear to have a significant impact on the length of stay. This correlation is observed in the Spanish registry as well [16]. This highlights a key area for improvement within the Greek healthcare system, prioritising timely surgical intervention as a strategy to reduce hospitalisation duration.

Limitations

Although this study represents the largest Greek cohort to date in terms of patient numbers, the total sample (just over 2,000 patients) remains relatively small when compared to other national registries. The participating hospitals were scattered across the country, mostly located in major cities, but still represent only a small fraction of the national healthcare system. Including a broader and more diverse population would provide a more accurate reflection of the overall Greek healthcare landscape. This study is subject to potential selection bias due to the non-random recruitment of participants, an inherent limitation of all registry studies. Additionally, the findings may have limited external validity, as the sample was drawn from a small number of hospitals, which may differ from other populations in demographic or clinical characteristics. The high incidence of delayed surgeries and their evident impact on length of stay highlight the need to investigate the underlying causes. Addressing these delays presents a clear opportunity for improvements in the national healthcare system.

## Conclusions

The introduction of Greece's first national hip fracture registry demonstrates its value as an important tool for assessing the current condition of the country’s healthcare system. Its implementation highlights a systemic shortcoming. Specifically, the low proportion of patients undergoing timely surgical intervention, which leads to significantly prolonged hospital stays. Nevertheless, the 30-day mortality rate remains comparable to that of other European countries. Notably, the high percentage of patients living independently before the injury, along with the significant number who return home after hospitalization, appears to have a positive impact on mortality outcomes.
